# Q-TOF LC-MS compounds evaluation of propolis extract derived from Malaysian stingless bees, *Tetrigona apicalis*, and their bioactivities in breast cancer cell, MCF7

**DOI:** 10.1016/j.sjbs.2022.103403

**Published:** 2022-08-05

**Authors:** Wan Ahmad Syazani Mohamed, Noor Zafirah Ismail, Musthahimah Muhamad, Eshaifol Azam Omar, Nozlena Abdul Samad, Jer Ping Ooi, Sharlina Mohamad

**Affiliations:** a Centre for Coordination of Clinical Research Network (CCRN), Block B4, Institute for Clinical Research (ICR), National Institutes of Health (NIH), Jalan Setia Murni U13/52, Seksyen U13, Setia Alam, 40170 Shah Alam, Selangor, Malaysia; b Centre for Clinical Trial (CCT), Block B4, Institute for Clinical Research (ICR), National Institutes of Health (NIH), Jalan Setia Murni U13/52, Seksyen U13, Setia Alam, 40170 Shah Alam, Selangor, Malaysia; c Department of Toxicology, Advanced Medical and Dental Institute (IPPT) Universiti Sains Malaysia, SAINS@BERTAM, 13200 Kepala Batas, Penang, Malaysia; d Breast Cancer Translational Research Program (BCTRP@IPPT), Advanced Medical and Dental Institute (IPPT), Universiti Sains Malaysia, SAINS@BERTAM, 13200 Kepala Batas, Penang, Malaysia

**Keywords:** *Tetrigona apicalis*, Propolis, LC-MS, Antioxidant, TPC, TFC, Apoptosis, MCF7

## Abstract

Propolis is known to exhibit various phytochemical compounds that aid in several biological activities. The current study investigates the phytochemical compounds of ethanolic extract of propolis of *Tetrigona apicalis* (EEP) using Q-TOF LC-MS, its antioxidant properties using DPPH and ABTS^+^ radical scavenging assays, total phenolic (TPC) and flavonoid content (TFC), using Folin-Ciocalteu and Aluminium Chloride method, respectively, as well as proapoptotic effects, based on the selected IC_50_ of the cytotoxic study conducted for EEP using annexin V-FITC assay. Terpene and polyphenol were among of 17 identified compounds. The EC_50_ of EEP for DPPH and ABTS^+^ was 1.78 mg/mL and 1.68 mg/mL, while the EEP exhibited TPC and TFC values of 31.99 mgGAE/g and 66.4 mgQCE/g, respectively in which the parameters were strongly correlated. The IC_50_ of EEP effectively induces apoptosis in MCF7 cells. In conclusion, EEP possessed important phytochemical compounds that work excellently as antioxidants and anticancer agents.

## 1 Introduction

Propolis has long been used as a part of herbal medicine in several countries. This traditional herbal medicine is made from sticky, resinous materials from various plant sources that served as a “glue” to repair any cracks or holes in the bee’s nest. Propolis, which is derived from the Greek word, “pro-” (in defense or barrier) and “polis-” (city), has been used as a medical remedy since at least 300 BCE to treat several diseases such as abscesses and cold sores (Ghisalberti 1979). Several factors, such as sources or exudates of plant materials, secretion of substances from bee’s metabolism, and geographical climates, may contribute to the diversity of biological compounds in propolis (Mat Nafi et al. 2019). Therefore, these bioactive properties are used in several studies to conclude its action in antioxidant, anti-inflammatory, antibacterial, antiviral, antifungal, antihepatotoxic, and anticancer activities (Ibrahim et al., 2016, Awang et al., 2018).

Propolis research has been widely discussed in Southeast Asian countries, particularly in Malaysia, for its beneficial pharmacological properties. For instance, the detection of phytochemical compounds using gas chromatography-mass spectrometry (GC–MS), quadrupole time-of-flight liquid chromatography-mass spectrometry **(**Q-TOF LC-MS), thin-layer chromatography (TLC) along antioxidant activity was carried out in some studies (Ibrahim et al., 2016, Zhao et al., 2017, Mohd et al., 2018). However, studies on the anticancer activities of Malaysian propolis are still scarce, with only a few cell lines were investigated such as SK-UT-1 (uterine leiomyosarcoma cells), HeLa (cervical cancer cells), and MDA-MB-231 (breast cancer cells) (Gapar, 2018, Mat Nafi et al., 2019). Nevertheless, the other specific biological benefits of each stingless bee species have yet to be explored, as there are 17 to 32 well-known species of stingless bees in Malaysia (Kelly et al. 2014).

The current study is a continuation of a previous study on *Tetrigona apicalis* propolis extract conducted by Mohamed et al. (2020). *T. apicalis* was discovered in Southeast Asia, Indo-Malaya/Australasia, and particularly in the subtropical regions (Rasmussen 2008). This species, as one of the three most common species of Malaysian stingless bees, is native to the wild, as opposed to the other two species, *Heterotrigona itama,* and *Geniotrigona thoracica*, which are kept for beekeeping/meliponiculture (Kelly et al. 2014). This characteristic has resulted in *T. apicalis* being a potent pollinator group in most ecosystems, especially in Malaysian virgin jungle reserves (Jauker et al., 2012, Salim et al., 2012).

Up to this point, there have been no studies that have focused on the screening of bioactive compounds using Q-TOF LC-MS with the proapoptotic potential of *T. apicalis* propolis extract towards hormone-responsive breast cancer, MCF7. Thus, this study aims to identify potential phytochemical compounds in *T. apicalis* propolis extract using Q-TOF LC-MS and evaluates its antioxidant properties, along with total phenolic and flavonoid content, in which these components may contributes to anticancer activities. Additionally, the present study also uses IC_50_ values from cytotoxic activity as described by Mohamed et al. (2020) to conduct apoptosis induction assay. The findings of the current study will hypothetically demonstrate the potential of T. apicalis propolis extract to be a potent anticancer agent, particularly for hormone-responsive breast cancer in the near future.

## 2 Materials and methods

### 2.1 Materials

The analytical grade (dimethyl sulfoxide (DMSO), ethanol, and methanol), powder form of 2,2-diphenyl-1-picrylhydrazyl (DPPH), 2,2′-azino-bis-3-ethylbenzothiazoline-6-sulfonic acid (ABTS^+^), potassium persulfate (K_2_S_2_O_8_), sodium carbonate (Na_2_CO_3_), sodium nitrite (NaNO_2_), aluminum chloride (AlCl_3_), sodium hydroxide (NaOH)), Folin-Ciocalteu reagent, standards for cytotoxic assay (tamoxifen), standard for antioxidant assays (quercetin, gallic acid and vitamin E analogue, 6-hydroxy 2,5,7,8-tetramethylchroman-2-carboxylic acid (Trolox) were purchased from Sigma Aldrich (St. Louis, Missouri, United States). The liquid chromatography solvent grades (formic acid and acetonitrile) were purchased from Qrec (Rawang, Selangor, Malaysia). Roswell Park Memorial Institute (RPMI-1640), Dulbecco’s Modified Eagle Medium (DMEM), 0.25 % trypsin/ ethylenediaminetetraacetic acid (EDTA), fetal bovine serum (FBS), penicillin–streptomycin, phosphate buffer saline (PBS), horse serum, hydrocortisone, insulin, epidermal growth factor (EGF), and 3-(4,5-dimethylthiazol-2-yl)-2,5-diphenyltetrazolium bromide (MTT) were purchased from Gibco (Waltham, Massachusetts, United States). The 5 × Annexin-V binding buffer, fluorescein isothiocyanate (FITC) Annexin-V, and propidium iodide (PI) staining solution were acquired from Invitrogen (Waltham, Massachusetts, United States).

### 2.2 Sample collection and identification of T. Apicalis species

*T. apicalis* propolis was collected at Tanjung Malim, Perak, Malaysia (GPS coordinate 3°73ʹ07.56′′ N, 101°55ʹ37.26″ E). The inner part of the bee nest was collected using the method described by Bonamigo et al. (2017) with minor modifications. The bee samples were collected from the hive to determine the stingless bee species. The bee sample was placed into the killing jar (consisting of 70 % isopropyl alcohol soaked in alcohol swabs along with a few drops of 5 % glacial acetic acid) and tightly closed. The specimen container that contained silica gel was ready for the dead bees to be identified. Finally, the specimen identification was done by the Centre for Insect Systematics (CIS), School of Environmental and Natural Resource Sciences, Faculty of Science and Technology, Universiti Kebangsaan Malaysia (UKM).

### 2.3 Preparation of propolis extracts

The sample was prepared using a modified version of the method described by Kothai and Jayanthi (2014). The sample of *T. apicalis* propolis was prepared in a powdered form and approximately measured for 10 g. The propolis was then extracted using 80 % ethanol and continuously stirred at 400 rpm using an orbital shaker (Buch & Holm, Hovedstaden, Denmark) for 24 h to obtain the crude extract. The sample suspensions were separated by centrifugation at 3000 rpm for 10 min and the extract was then filtered using filter paper. Subsequently, the ethanol was removed by using a rotary evaporator (Buchi, Flawil, Switzerland). The ethanolic extract of propolis (EEP) was later stored in a − 20 °C freezer before being freeze-dried to powder form.

### 2.4 Quadrupole time-of-flight liquid chromatography-mass spectrometry (Q-TOF LC-MS) analysis

The Q-TOF LC-MS analysis of *T. apicalis* propolis extract was done using Agilent 1290 Infinity LC System coupled to Agilent 6520 Accurate-Mass-Q-TOF mass spectrometer with positive and negative mode electrospray ionization (ESI). Agilent ZORBAX SB-C18 column was used to conduct the separation with the diameter of 2.1 mm, length of 150 mm, particle size of 3.5 µm with an operating temperature of 25 °C. The condition setting was established in a mobile phase of (A) 0.1 % formic acid in distilled water and (B) 0.1 % formic acid in acetonitrile; the gradient-elution were corresponded as 95 % A and 5 % B (0.00 min), 95 % A and 5 % B (0.00 – 5.00 min), 0 % A and 100 % B (5.00 – 20.00 min), 0 % A and 100 % B (20.00 – 25.00 min with injection volume of 1.0 µL and flow rate of 0.5 mL/min. The major operating parameters for the Q-TOF were set as follows: drying temperature of 300 °C, nebulizer pressure of 45 psig, drying gas 10 L/min, and capillary voltage 4.0 kV. The data acquisition was set to 100–3200 *m*/*z* at a rate of 1.0 spectrum/ms to briefly screen the compounds found in EEP. Agilent MassHunter Qualitative Analysis software was used to process the data. The compounds were chosen based on the comparison from METLIN metabolite and the chemical entity database. The compounds with an 80 % likeliness to chemical compounds from METLIN were selected for the present study. To validate the findings of this study, the identified compounds were screened and compared to standard compounds of Malaysian propolis as proposed by Zhao et al.(2017).

### 2.5 DPPH radical scavenging assay

The DPPH radical scavenging activity in EEP was determined using the method described by Brand-Williams et al. (1995) with some modifications. About 0.6 mM methanolic solution of DPPH was prepared to serve as the working solution with a target absorbance of 1.1 (±0.02) at 517 nm using a spectrophotometer (Biomate spectrophotometer, Thermo Fisher Scientific, USA).

The samples were prepared in various concentrations ranging from 0.02 to 0.313 mg/mL and diluted with 1 mL of methanol. Then, 150 µL of DPPH working solutions were mixed with 7.5 µL of samples in a 96-well plate and placed in the dark for 30 min at room temperature, whereas methanol was used as blank. All concentrations were measured in triplicate. The positive control (Trolox) was treated in the same conditions as the samples. The scavenging effect percentage was determined by using the equation below:

(1) $$Inhibition\left(\%\right)=\frac{A_{1}-A_{2}}{A_{1}}\times 100{\%}^{15}$$

where A_1_ is the absorbance of the control and A_2_ is the absorbance of the samples. The mean half-maximal response of EEP concentration (EC_50_) value was estimated as mean ± standard deviation (SD). The EC_50_ for DPPH for both EEP and Trolox was calculated using four-parameter logistic regression equation calculator by AAT Bioquest (Sunnyvale, USA) (Costales-Carrera et al. 2019).

### 2.6 ABTS^+^ radical scavenging assay

The ABTS^+^ radical scavenging activity in EEP was evaluated using the method explained by Vongsak et al., 2015, Campos et al., 2015 with slight modifications. About 7 mM ABTS^+^ aqueous solution and 2.45 mM potassium persulfate in water were prepared and mixed. Subsequently, the mixture was placed in the dark for 12 to 16 h at room temperature to yield a stock solution. The ABTS^+^ radical solution was prepared by reacting 1 mL ABTS^+^ radical with 50 mL methanol to achieve an absorbance of 0.70 (±0.02) at 734 nm using a spectrophotometer.

Samples with concentrations ranging from 0.02 to 0.313 mg/mL were prepared. About 1.25 µL of samples were allowed to mix with 125 µL of ABTS^+^ radical in a 96-well plate. The mixture was kept in the dark at 37 °C for 6 min, whereas methanol was used as blank. All concentrations were repeated in triplicates. The positive control, Trolox, was used in the same setting as the samples. The percentages of scavenging effects were calculated by the equation below:

(2) $$Inhibition\left(\%\right)=\frac{A_{1}-A_{2}}{A_{1}}\times 100\%$$

where A_1_ is the absorbance of the control, and A_2_ is the absorbance of the samples. The EC_50_ value was calculated as mean ± SD. The EC_50_ for ABTS^+^ was calculated using linear regression equations, in which for EEP is y = 142.28x + 6.0714 (R^2^ = 0.9928) and Trolox is y = 29.721x (R^2^ = 0.9809).

### 2.7 Total phenolic compounds (TPC)

Total phenolic content (TPC) was determined by using the Folin-Ciocalteu method with slight modifications (Kothai and Jayanthi 2014). The samples with various concentrations ranging from 0.1 to 12.5 mg/mL were initially dissolve with 1 mL of methanol, and 12 µL of each sample was allowed to mix with 60 µL of 0.2 mol/L Folin-Ciocalteu solution for 10 min. Then, the mixture was added with 48 µL Na_2_CO_3_. The samples were incubated for 30 min at room temperature. The absorbance was measured using a spectrophotometer at 760 nm, with gallic acid serving as a standard reference. The TPC of EEP was signified as gallic acid equivalent (GAE) in µg/g dry weight of the extract using standard curve valued 12.5 to 100 µg/mL. The regression curve for TPC (with gallic acid as a standard) comprises the equation y = 0.0022x + 0.0103, with R^2^ = 0.9992.

### 2.8 Total flavonoid compounds (TFC)

The total flavonoid contents (TFC) were determined using the Aluminium Chloride Method with some modifications (Fidrianny et al. 2015). The samples with various concentrations from 0.1 to 12.5 mg/mL were initially dissolved with 1 mL of methanol. An amount of 10 µL of each sample was then allowed to react with 3 µL and 40 µL of NaNO_2_ and distilled water, respectively. About 3 µL of 10 % AlCl_3_ was added after 5 min, followed by 20 µL of sodium hydroxide solution after the former solution was mixed had been mixed for 5 min. The absorbance was read using a spectrophotometer at 420 nm with quercetin as a standard reference. The TFC of EEP was expressed as quercetin equivalent (QCE) in µg/g dry weight of the extract using standard curve valued 6.25 to 100 µg/mL. The regression curve for TFC (with quercetin as a standard) comprises the equation y = 0.0157x – 0.0675, with R^2^ = 0.9959.

### 2.9 Quantification of apoptosis induction

The Annexin V-FITC assay was carried out using an apoptosis kit (Invitrogen, Waltham, Massachusetts, United States) according to the protocols provided in the kit. Briefly, MCF7 cells were separately treated using the IC_50_ value of EEP and the positive control, Tamoxifen (32.70 µg/mL and 7.85 µg/mL, respectively), and incubated for 72 h following the selected IC_50_ value of cytotoxic assay by Mohamed et al. (2020). Then, the cells were washed by using cold PBS and centrifuged to obtain the cell pellet. The supernatant was removed, and the cell pellet was resuspended with 1X Annexin-V binding buffer to determine the cell density of 2.5 × 10^6^ cells/mL. Then, 5 µL of FITC Annexin-V and 1 µL of 100 µg/mL PI working solution were added to each 100 µL of cell suspension. The cells were incubated in the dark at room temperature for 15 min. After incubation, a 400 µL 1X Annexin-V binding buffer was added to the tubes. The tubes were kept on ice prior to the apoptosis analysis.

The stained cells were analyzed by using a flow cytometer (FACS Calibur – Becton Dickinson, USA) with fluorescence emission measurements of 530 nm and greater than 575 nm. Each data set contained 10 000 cells for analysis. The observation and identification of cells populations were divided into four cell groups: 1) viable cells: negative Annexin V and PI; 2) early apoptosis: positive Annexin V and negative PI; 3) late apoptosis: positive Annexin V and PI; 4) necrotic or dead cells: negative Annexin V and positive PI. The untreated cells served as a negative control. To assess the apoptotic flow of IC_50_ across the three timeframes of incubation, the incubation duration of chosen IC_50_ and untreated cells was also analyzed and compared with the other two time periods (24 h and 48 h).

### 2.10 Statistical analysis

The statistical analysis was done in three replicates, and the data were evaluated as mean values with standard deviation (SD), with p-values < 0.05 considered significant. The percentage of apoptosis induction for selected IC_50_ of EEP and Tamoxifen was calculated using an independent sample *t*-test, whereas the relationship between antioxidant activity with TPC and TFC was calculated using Pearson’s correlation coefficient. Both calculations were done using International Business Machine Corporation Statistical Product and Service Solutions (IBM SPSS) Statistics Version 27.

## 3 Results

### 3.1 Extraction of T. Apicalis propolis extract

The yield (%) of the crude extract along with its physical appearance was deliberated and documented. The crude extract was whitish in appearance, and the samples were in powder form. The EEP sample that was derived from crude ethanolic extract produced a yield of 57 %.

### 3.2 Q-TOF LC-MS analysis

The detection of phytochemical compounds in the extract using Q-TOF LC-MS was analyzed on EEP with separation of major bioactive compounds using LC and identification via MS with positive and negative mode ESI. The score of similarities was supported by the molecular feature extraction (MFE) algorithm and molecular formula generator (MFG) software. Using at least 80 % similarities with chemical compounds from the METLIN library, the results of Q-TOF LC-MS analysis are summarized in Table 1(a) and Table 1(b).

**Table 1a:** Phytochemical compounds identified in EEP using Q-TOF LC-MS (Positive ESI).

Peak	Rt	*m*/*z*	Error (ppm)	Formula	MW (g/mol)	Identification	MS/MS (*m*/*z*)	Score (MFE)	Score (MFG)
1	16.359	205.1952	−0.47	C_15_ H_24_	204.1879	(S)-beta-himachalene	203.1849	100	83.77
2	11.095	221.1904	−1.84	C_15_ H_24_ O	220.1831	Ishwarol	195.1408, 203.1824, 209. 1542	100	85.77
3	16.708	279.1692	2.79	C_15_ H_22_ N_2_ O_3_	278.1623	Leucyl-phenylalanine	279.1661	100	91.44
4	16.723	299.2102	−3.05	C_17_ H_28_ N_2_ O	276.221	Etidocaine	279.1661	100	80.2
5	15.720	315.204	−3.90	C_14_ H_26_ N_4_ O_4_	314.1966	Prolyl-alanyl-lysine	297.1923	100	91.37
6	15.720	397.2067	−0.34	C_17_ H_32_ O_10_	396.1997	1-Hexanol arabinosylglucoside	392.2495	100	91.19
7	19.002	427.366	−3.10	C_23_ H_46_ N_4_ O_3_	426.3583	*N*-stearoyl arginine	409.3548	100	85.87
8	16.778	469.3316	0.99	C_30_ H_44_ O_4_	468.3235	Ganoderic acid DM	441.3594, 455.3560	100	85.9
9	22.176	493.339	1.66	C_29_ H_46_ N_2_ O_3_	470.3501	*N*-stearoyl tryptophan	441.3653, 455.3663, 471.3587, 481.4798	100	93.73
10	17.434	497.3722	−1.86	C_29_ H_50_ N_2_ O_3_	474.383	dl-*threo*-1-Phenyl-2-palmitoylamino-3-morpholino-1-propanol	471.3541, 483.3547	97.8	96.66
11	15.025	531.3682	−2.08	C_32_ H_50_ O_6_	530.3618	Acinospesigenin A	503.3394, 513.3530	80	87.23
12	15.719	771.4169	−4.46	C_39_ H_62_ O_15_	770.4123	Scopoloside II	762.6281	100	83.58

Note: RT, retention time; MW, molecular weight; MFG, molecular formula generator; MFE, molecular feature extraction.

**Table 1b:** Phytochemical compounds identified in EEP using Q-TOF LC-MS (Negative ESI).

Peak	Rt	*m*/*z*	Error (ppm)	Formula	MW (g/mol)	Identification	MS/MS (*m*/*z*)	Score (MFE)	Score (MFG)
1	14.311	331.1977	3.15	C_14_ H_28_ N_4_ O_5_	332.2049	Valine-serine-lysine	331.1975	100	92.41
2	15.717	419.2137	0.91	C_26_ H_29_ F N_2_ O_2_	420.2209	Levocabastine	409.1841	100	98.21
3	9.612	515.1704	1.02	C_30_ H_28_ O_8_	516.1779	Rottlerin	491.9059, 505.1307	100	98.45
4	16.722	631.3827	4.63	C_36_ H_56_ O_9_	632.3895	Oleanolic acid 3-O-beta-d-glucosiduronic acid	631.3918	100	85.4
5	15.718	747.4208	−1.14	C_16_ H_30_ N_4_ O_6_	374.217	Leucine-aspartate-lysine	373.2126, 419.2169, 567.3460, 641.3202	100	95.8

Note: RT, retention time; MW, molecular weight; MFG, molecular formula generator; MFE, molecular feature extraction.

The phytochemical compounds of propolis previously identified in several studies were used as external standards in order to accentuate the findings of the extract (Midorikawa et al., 2001, Carvalho et al., 2011). A study on Malaysian *H. itama* propolis extract conducted by Zhao et al. (2017) discovered the presence of several important bioactive compounds, such as gallic acid, kaempferol, and caffeic acid. Thus, the standards as described by Zhao et al. (2017) were incorporated in the screening of the present study.

Based on Table 1(a) and Table 1(b), 17 compounds have been identified, with 12 compounds from positive ESI and 5 compounds from negative ESI. The LC-MS also detected two compounds that had the same retention time but different *m*/*z*. At retention time 15.720 in positive ESI, prolyl-alanyl-lysine and 1-hexanol arabinosylglucoside were identified with each fragmentation ions of 297.1923 *m*/*z* (with loss of 17 g/mol) and 392.2495 *m*/*z* (with loss of 3 g/mol), respectively. The ion fragmentation of prolyl-alanyl-lysine corresponds to the loss of NH_3_ of tripeptide (Zhang et al. 2019). however, for 1-hexanol arabinosylglucoside, the identified peak was most likely not due to molecular ion peak, as the fragmentation peaks were in the range of 3–14 mass units from the suggested peak that could result from the loss of up to 3 hydrogen atoms (Dunnivant and Ginsbach 2011). For the current study, EEP did not exhibit any compounds that were matched with external standards. The significance of the identified compounds will be explained later in the discussion section.

### 3.3 Determination of DPPH and ABTS^+^ radical scavenging activity

The DPPH and ABTS^+^ radical scavenging activities of EEP were determined for several concentrations to signify the presence of potential antioxidant activities. Table 2 lists the calculated concentration values of EEP needed to scavenge DPPH and ABTS^+^ by half (EC_50_). Because EEP and Trolox both used the same concentration range, the maximum DPPH and ABTS^+^ radical scavenging activities of EEP were at 0.313 mg/mL with 3.59 % and 9.5 % inhibition in correlation to 92.5 % Trolox and 49.8 % Trolox, respectively. On contrary, the lowest EEP radical scavenging activity was at 0.02 mg/mL with no inhibition and 1.2 % inhibition corresponding to 27.5 % Trolox and 7.8 % Trolox, for DPPH and ABTS^+^, respectively. The EC_50_ of EEP for DPPH and ABTS^+^ were 1.78 mg/mL and 1.68 mg/mL, respectively, whereas the EC_50_ of Trolox for DPPH and ABTS^+^ were 0.04 mg/mL and 0.31 mg/mL, respectively.

**Table 2:** The concentrations of EEP and Trolox with DPPH and ABTS + radical scavenging activity and its corresponding EC_50._

Concentration (mg/mL)	EEP Radical Scavenging Activity		Trolox Radical Scavenging Activity	
	**DPPH**	**ABTS^+^**	**DPPH**	**ABTS^+^**
0.02	–	1.2 %	27.5 %	7.8 %
0.313	3.59 %	9.5 %	92.5 %	49.8 %
EC_50_	1.78 mg/mL	1.68 mg/mL	0.04 mg/mL	0.31 mg/mL

### 3.4 Determination of TPC and TFC

The phenolic and flavonoid contents are noted to play a significant role for antioxidant activities, particularly in propolis (Miguel et al. 2010). By using the same concentration for both tests, Table 3 shows the highest concentration of EEP (12.5 mg/mL) to exhibit total phenolic and flavonoid contents, with TPC valued at 31.99 mgGAE/g and TFC valued at 66.4 mgQCE/g.

**Table 3:** The value of EEP concentration for with its maximal TPC and TFC. Data are mean ± SD of triplicate experiments.

	**TPC (mgGAE/g)**	**TFC (mgQCE/g)**
Linear Regression Equation	y = 0.0022x + 0.0103	y = 0.0157x – 0.0675
R^2^ value	0.9992	0.9959
EEP	31.99 ± 0.01	66.40 ± 0.01

### 3.5 Correlation of DPPH, ABTS^+^, TPC and TFC

The relationship between antioxidant activity with TPC and TFC was measured using the Pearson correlation coefficient, in which the correlation coefficient ranged from + 1 to − 1. Based on Table 4, there is a strong positive relationship between antioxidant activity with TPC and TFC, with all correlations falling between r = 0.950 and r = 0.971.

**Table 4:** The Pearson’s correlation coefficient (r) of DPPH, ABTS^+^, TPC and TFC. The statistical difference was calculated using Student’s paired *t*-test. All are significant with p value < 0.05.

	TPC	TFC
DPPH	0.950	0.961
ABTS^+^	0.971	0.956

### 3.6 Apoptosis induction assay of EEP

**Fig. 1:**
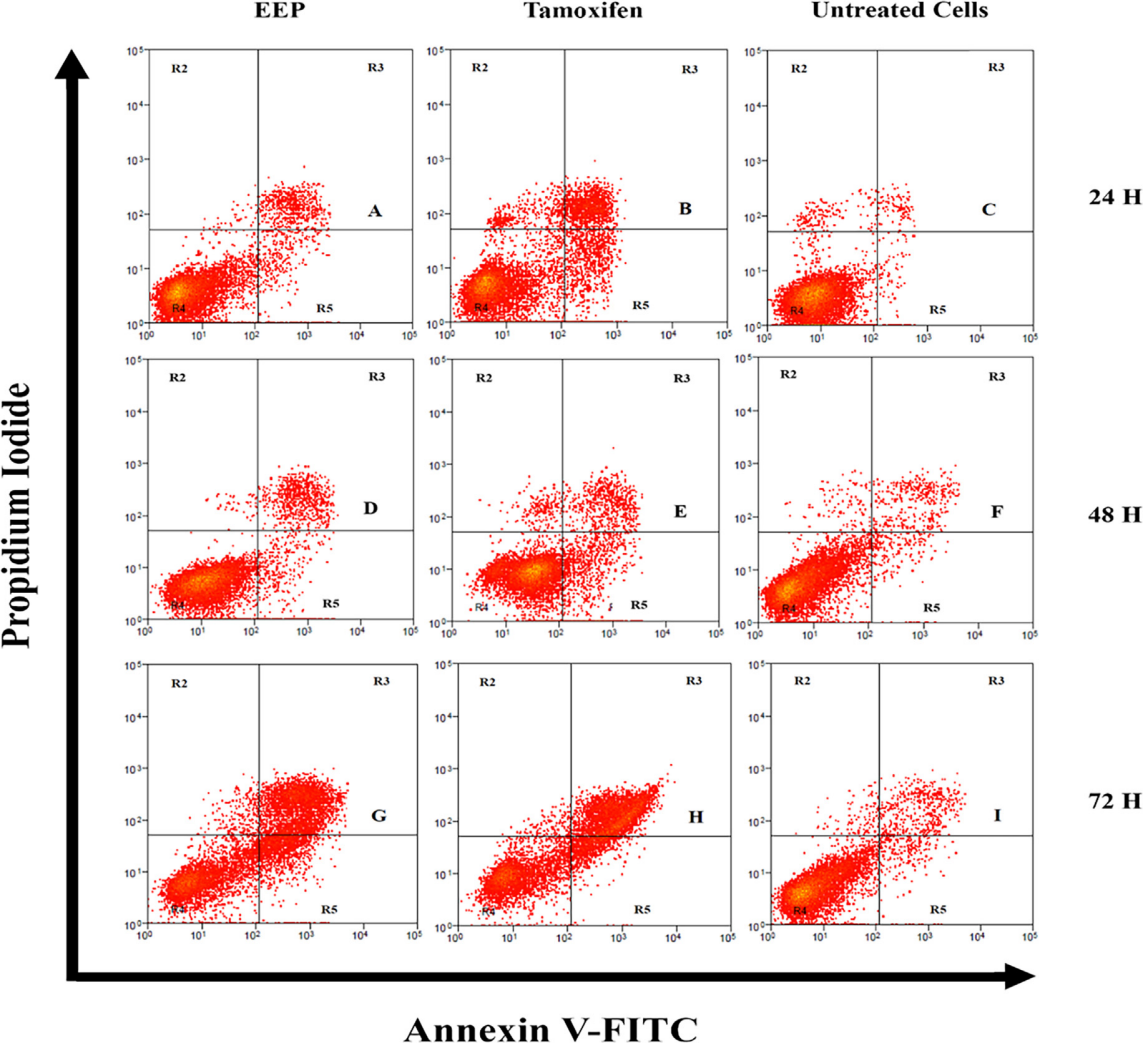
Flow cytometry analysis of selected IC_50_ for MCF-7 cells treated with EEP, Tamoxifen and untreated cells for 24 h (A, B and C), 48 h (D, E and F) and 72 h (G, H and I). The results were summarized for three independent experiments. For each panel, the viable cells are shown in the lower left quadrant (R4), early apoptosis in lower right quadrant (R5), late apoptosis in right upper quadrant (*R*3) and necrosis in the upper left quadrant (R2).

The percentage of apoptotic cells was determined using a flow cytometer, in which staining (consisted of annexin V and PI) was done to MCF7-treated with EEP, MCF7-treated with tamoxifen, and untreated cells. Table 5 shows the percentage of apoptosis induction of selected IC_50_ for MCF7-treated with EEP (32.70 µg/mL), MCF7-treated with tamoxifen (7.85 µg/mL) and untreated cells in three incubation points, while Fig. 1 is the flow cytometry analysis of IC_50_ of MCF7 and tamoxifen with untreated cells in 3 different incubation period (Mohamed et al. 2020). Based on Table 5 and Fig. 1, the apoptosis induction assay validates the cytotoxic study of selected IC_50_ conducted by Mohamed et al. (2020), in which the cell viability of viable, early apoptosis, late apoptosis, and necrotic/dead cells corresponded to 48.39 ± 2.06 %, 14.02 ± 0.98 %, 35.25 ± 1.16 %, and 2.34 ± 0.14 %, respectively.

**Table 5:** The percentage of apoptosis induction of selected IC_50_ of EEP, Tamoxifen with untreated cells in three incubation points. Values are presented as means ± SD of triplicate experiments. The statistical analysis was estimated using independent sample *t*-test for EEP and Tamoxifen in comparison to untreated cells.

Incubation Point (h)	Cell Viability (%)			
	**Cell Viable**	**Early Apoptosis**	**Late Apoptosis**	**Necrotic Cells**
MCF7 Treated with EEP				
24	88.20 ± 1.51**	4.09 ± 0.85*	7.26 ± 1.12**	0.45 ± 0.45*
48	87.58 ± 1.01**	3.90 ± 0.31**	7.53 ± 0.51**	0.99 ± 0.26
72	48.39 ± 2.06**	14.02 ± 0.98**	35.25 ± 1.16**	2.34 ± 0.14**
MCF7 Treated with Tamoxifen				
24	81.9 ± 0.57**	9.93 ± 0.23**	6.18 ± 1.06**	1.99 ± 0.72
48	72.75 ± 0.79**	8.79 ± 0.25**	15.88 ± 0.35**	2.58 ± 0.28**
72	41.67 ± 1.99**	10.33 ± 1.16**	46.64 ± 1.83**	1.36 ± 0.25
Untreated Cells				
24	96.65 ± 0.43	0.92 ± 0.21	0.90 ± 0.08	1.50 ± 0.19
48	93.74 ± 0.66	1.79 ± 0.19	3.41 ± 0.52	1.06 ± 0.12
72	92.14 ± 0.66	2.16 ± 0.19	4.68 ± 0.52	1.02 ± 0.12

Note: *, p value < 0.05; **, p value < 0.01.

## 4 Discussions

Overall, the detected compounds in EEP for positive ESI of Q-TOF LC-MS mostly consisted of terpene groups. Based on Table 1(a), both (S)-beta-himachalene and ishwarol belong to terpene derivatives of sesquiterpene hydrocarbon and oxygenated sesquiterpenes, respectively. Both types of sesquiterpenes have been mentioned in several studies for their potential to act as potent antioxidant and anticancer agents (Khan et al., 2008, Dahham et al., 2015, Jain et al., 2016). Xia et al. (2020) also reported that ganoderic acid DM (which belongs to triterpenoid) proves to induce autophagy apoptosis in non-small cell lung carcinoma via inhibition of PI3K/Akt/mTOR pathway. Aside from that, Tyler et al. (2015) discovered that dl-*threo*-1-phenyl-2-palmitoylamino-3-morpholino-1-propanol (dl-PPMP) potentiates cisplatin cytotoxicity in acquired cisplatin-resistance of lung carcinoma and malignant pleural mesothelioma.

In terms of negative mode ESI of Q-TOF LC-MS, however, only one triterpenoid was present, which was oleanolic acid 3-O-beta-d-glucosiduronic acid. The report is similar to those of Saleem et al. (2020), as this compound was present in the methanolic extract of *Bougainvillea glabra* flowers. However, the compound’s ability to act as an antioxidant and anticancer agent had yet to be discovered. Rottlerin, a polyphenol compound, was also discovered in the present study. Chhiber et al. (2016) demonstrated rottlerin’s ability to act as an antioxidant, as it reduced NADPH oxidase activity, inhibited dysfunction of mitochondria, and maintained antioxidant condition.

To the best of our knowledge, this is the first time the bioactive compounds are discovered for Malaysian propolis. As since this study only focuses on phytochemical screening using Q-TOF LC-MS to illustrate the therapeutic significance of compounds from crude EEP, the selection and evaluation of potential compounds will need to be specified further with fractionation using polar and non-polar solvents, and isolation from the pure fraction with thin layer chromatography (TLC) and column chromatography.

In relation to antioxidant activities, based on the comparison of EC_50_ in Table 2, it is also concluded that both radical scavenging activities of DPPH and ABTS^+^ corresponded with the concentration gradient (concentration-dependent effect). The current findings are also supported by multiple studies that agreed on the presence of antioxidant activities in *T. apicalis* propolis extract (Rosli et al., 2017, Asem et al., 2019). By using at least 80 % of compound similarities in Q-TOF LC-MS, it can be noted that both terpene and polyphenol contributed to the antioxidant activities in EEP. However, there is a lack of a definitive or optimized method to measure total terpene content in EEP because terpene constituents are the largest group of natural compounds (Indumathi et al. 2014). Therefore, the current study only focusing to determine the total phenolic content. In addition, as flavonoid is the largest subclass group of polyphenols, the measurement of total flavonoid content was also done in the current study.

Based on the TPC and TFC results in Table 3, it is noted that the value of TFC is greater than the TPC value. This finding is in agreement with several studies, which included propolis extract of Malaysian stingless bees, *T. apicalis* and *H. itama* (Rosli et al., 2017, Awang et al., 2018). According to Katsube et al., 2004, Wu et al., 2004, the most likely reason is phenolics characterized by all compounds that contained a phenolic group (monophenol, diphenol, triphenol, or polyphenol). Due to the vast complexity of compounds in phenolics, the characterization of each compound with its structure elucidation can be difficult, especially when dealing with many herbal extracts. According to Anokwuru et al. (2011), depending on the number of phenolic groups in phenolic compounds, the response towards Folin-Ciocalteu reagent might react differently. Thus, the slightly lower value in TPC in this study did not reflect the total actual value of phenols in EEP.

Additionally, as though the findings of polyphenol using Q-TOF LC-MS of the current study was only rottlerin, the contribution by other types of polyphenols in antioxidant activities may be also contributed by the polyphenols that valued < 80 % similarities from METLIN library. Nevertheless, there is a strong relationship between antioxidant activity with TPC and TFC, with all correlations falling between r = 0.950 and r = 0.971. Table 4 shows that the correlation between antioxidant activities with TPC and TFC is all strongly positive, with r-values greater than 0.9.

Based on Table 5 and Fig. 1, the apoptosis induction assay in this study validates the cytotoxic study of selected IC_50_ conducted by Mohamed et al. (2020), whereby the cell viability percentage of viable cells, early apoptosis, late apoptosis, and necrotic/dead cells corresponded to 48.39 ± 2.06 %, 14.02 ± 0.98 %, 35.25 ± 1.16 %, and 2.34 ± 0.14 %, respectively. In comparison to a study by Gapar (2018) for EEP of *T. apicalis*, whereby the cells percentage of early and late apoptosis phase of HeLa cells were 6.6 % and 23.97 %, respectively; the results for both phases in the current study was relatively higher, in which corresponded to 14.02 ± 0.98 % and 35.25 ± 1.16 %, respectively. Therefore, it was justified that EEP is more sensitive to cause apoptosis induction in early or late apoptosis in MCF7 than in HeLa cells. Thus, it can be concluded that the antioxidant capacities produced in EEP play a part to cause apoptosis induction in cancer cells. It is previously known that the antioxidants from plant origin with/without other natural sources have been shown to cause cell death through apoptosis induction in breast, lung, liver, colorectal, and alveolar cancers, in particular (Kntayya et al., 2018, Adebayo et al., 2019).

In relation to MCF7, it was reported that ganoderic acid DM that was found in the current study could induce DNA fragmentation and reduce the mitochondrial membrane potential in MCF7 cells, as reported by Wu et al. (2012). In addition, Torricelli et al. (2008) also reported that rottlerin was able to inhibit the nuclear factor κB/Cyclin-D1 cascade in MCF7, proving its anticancer activity. The molecular analysis, including the protein pathway using western blot analysis, will be recommended for future studies to confirm and validate the proteins responsible for activation of apoptosis cascade for EEP of *T. apicalis.*

## 5 Conclusion

In conclusion, EEP is proved to have significant bioactive compounds that was capable in various biological activities, including antioxidant and anticancer activities. Additionally, this study deduced that the apoptosis induction based on the selective IC_50_ of EEP conclusively signified the cytotoxic activity of EEP. The molecular validation using western blot analysis to conform the EEP apoptotic effect as well as compound fractionation and isolation may be recommended for further EEP studies.

## CRediT authorship contribution statement

**Wan Ahmad Syazani Mohamed:** Conceptualization, Methodology, Software, Formal analysis, Investigation, Writing – original draft, Visualization. **Noor Zafirah Ismail:** Data curation, Software, Investigation, Resources. **Musthahimah Muhamad:** Data curation, Software, Investigation, Resources. **Eshaifol Azam Omar:** Resources, Visualization, Project administration. **Nozlena Abdul Samad:** Resources, Visualization, Project administration. **Ooi Jer Ping:** Writing – review & editing. **Sharlina Mohamad:** Writing – review & editing, Supervision, Funding acquisition.

## Declaration of Competing Interest

The authors declare that they have no known competing financial interests or personal relationships that could have appeared to influence the work reported in this paper.
